# A pedunculated lymphangiomatous polyp of the palatine tonsil. A case report

**DOI:** 10.5935/1808-8694.20130069

**Published:** 2015-10-04

**Authors:** Betül Peker Cengiz, Mustafa Acar, Ersem Giritli

**Affiliations:** a Pathology M.D.; b Division of Otorhinolaryngology M.D.

**Keywords:** lymphangioma, palatine tonsil, polyps

## INTRODUCTION

The head and neck is the most common site of the lymphangiomatous lesions. Most arise in the skin and subcutaneous tissues, but other sites include the larynx, parotid gland, mouth, and tongue. Benign tumors of the tonsils are rarely seen compared to malignancies^1^, ^2^. These lesions have been described by different name in the literature such as angiomas^1^, ^3^, polypoid lymphangioma of the tonsil^4^, hamartomatous tonsillar polyp^4^, lymphoid polyp^5^, or tonsillar lymphangiomatous polyp^1^, ^4^, so it is difficult to determine the true incidence of these lesions.

In the literature less than 30 cases with lymphangiomatous polyp of the tonsils have been reported. The patients presented generally with dysphagia, sore throat or sensation of mass in the throat. The physical examination by indirect laringoscopy, unilateral tonsillary mass can be detected and these lesions are frequently misdiagnosed as malignancy^1^, ^4^, ^5^. The curative treatment is wide excision.

Here we presented a case of pedunculated lymphangiomatous polyp on palatine tonsil.

## CASE REPORT

A 36-year-old woman who was referred the Otolaryngology Department of Yunus Emre General Hospital, in May 2009 with difficulty of swallowing and foreign body sensation in the throat. She had complaints of sneezing which started two years ago and she had history of allergies. Physical examination of her oral cavity revealed a pale peduculated mass extending from the left palatine tonsil. The rest of oral cavity, nasopharynx and larynx were normal. There was no evidence of cervical lymphadenopathy. The mass was suspected as a benign polyp and intra-oral tumor excision was performed under local anesthesia.

**Figure 1:**
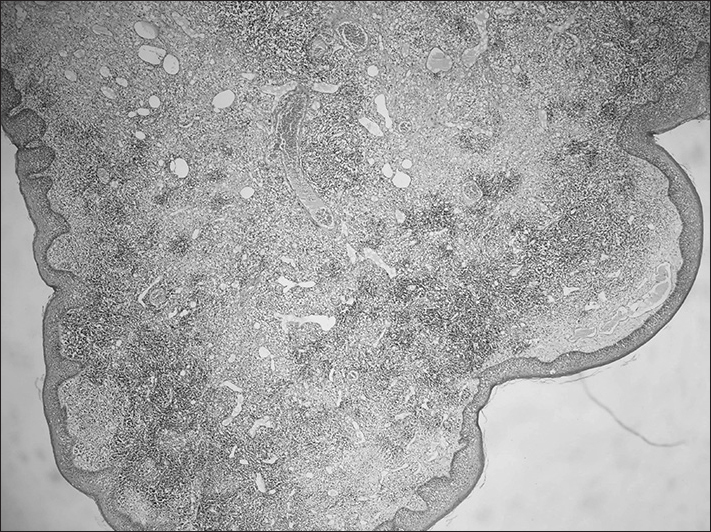
The three components of tonsillar lymphangiomatous polyps: dilated lymphatic channels, fibrous stroma, and lymphoid tissue (100x).

The specimen was measured macroscopically 1,3 x 0,7 cm in diameter. The mass was firm and smooth, with a small pedunculated base. Histologically, its surface was covered with parakeratotic squamous epithelium and, its stroma was composed of loose fibrouse tissue included numerous dilated lymphatic space and aggregates lymphoid tissue (Figure 1). In the light of these pathological findings the diagnosis of lymphangiomatous polyp was confirmed. After surgical excision, the patient remained recurrence free for 12 months of follow-up period.

## DISCUSSION

The head and neck is the most common site of the lymphangiomatous lesions. Most arise in the skin and subcutaneous tissues, but other sites include the larynx, parotid gland, mouth, and tongue^1^. In the literature in recent years, Gupta et al.^6^ described the lymphangiomatous polyp of the nasal cavity. Benign tumors or tumor-like lesions of the palatine tonsil are less common than malignant ones. Moreover, the tonsils are less common site for the development of pedunculated lymphangiomatous lesions^1^, ^2^. Lymphangiomatous polyp has been reported with the different names in the literature including angiomas^1^, ^3^, polypoid lymphangioma of the tonsil^4^, hamartomatous tonsillar polyp^4^, lymphoid polyp^5^, or tonsillar lymphangiomatous polyp^1^, ^4^ so it is difficult to determine the true incidence of these lesions. Kardon et al.^1^ reviewed the 26 cases of lymphangiomatous polyp of tonsils and he believed that lymphangiomatous tonsillar lesions have higher incidence than the reported cases in the literature.

Lymphangiomatous polyp usually occurs in young adult and children^1^. Kardon et al.^1^ reported the median age was 25.2 years in their study. Similarly, Barreto et al.^2^ also reported the median age of their patients was 29. The patients were generally presented with sore throat, dysphagia, dyspnea, and even a sense of mass depending on the size of the mass. On the other hand, the the patients might be asymtomatic, detection of these lesions might be incidental. Our patient presented with swallowing and foreign body sensation in the throat compatible with the literature.

The history and the clinical examination are important for the diagnosis, but histological examination is needed to establish the diagnosis. Some authors maintain the assertion that these lesions are most likely hamartomatous, because of a haphazard proliferation of stromal elements that are normally found in the tonsil^4^. Kardon et al.^1^ also reported that the idea of hamartomas origin of these lesions. Barreto et al.^2^ also reviewed the pathology of lymphangiomatous polyps displayed a wide spectrum of histological features, including varying amounts of fibrous and lymphoid tissues. Kardon et al.^1^ agreed with collagen and adipose tissue which were present in the stroma. Our cases of lymphangiomatous polyp contained loose fibrous connective tissue and rich lymphocyte infiltration with varying amounts of lympho-vascular proliferation.

Lymphangiomatous polyp should be considered in the differantial diagnosis of mass lesion in the tonsil^1^, ^4^, ^5^. The differential diagnoses should include papilloma, fibroepitelyal polyp, and lymphangioma^1^, ^4^.

Lymphangiomatous polyps of the tonsil are unusual benign hamartomatous lesions, and they are treated with curative intent by simple surgical excision^1^, ^4^. There have been no reported cases of disease recurrence or malignant transformation after excision.

## FINAL REMARKS

We think that lymphangiomatous polyps are more common than reported in the literature. However, the true incidence is not known because of different names are present in the literature. We believe that, our case is noteworthy to help the estimate of the true incidence in the future.
